# A Novel Multi-Antigen Virally Vectored Vaccine against *Mycobacterium avium* Subspecies *paratuberculosis*

**DOI:** 10.1371/journal.pone.0001229

**Published:** 2007-11-28

**Authors:** Tim J. Bull, Sarah C. Gilbert, Saranya Sridhar, Richard Linedale, Nicola Dierkes, Karim Sidi-Boumedine, John Hermon-Taylor

**Affiliations:** 1 Department of Cardiovascular Sciences-Surgery, St George's University of London, London, United Kingdom; 2 Wellcome Trust Centre for Human Genetics, Nuffield Department of Medicine, University of Oxford, Oxford, United Kingdom; Centre for DNA Fingerprinting and Diagnostics, India

## Abstract

**Background:**

*Mycobacterium avium* subspecies *paratuberculosis* causes systemic infection and chronic intestinal inflammation in many species including primates. Humans are exposed through milk and from sources of environmental contamination. Hitherto, the only vaccines available against *Mycobacterium avium* subspecies *paratuberculosis* have been limited to veterinary use and comprised attenuated or killed organisms.

**Methods:**

We developed a vaccine comprising a fusion construct designated HAV, containing components of two secreted and two cell surface *Mycobacterium avium* subspecies *paratuberculosis* proteins. HAV was transformed into DNA, human Adenovirus 5 (Ad5) and Modified Vaccinia Ankara (MVA) delivery vectors. Full length expression of the predicted 95 kDa fusion protein was confirmed.

**Principal Findings:**

Vaccination of naïve and *Mycobacterium avium* subspecies *paratuberculosis* infected C57BL/6 mice using DNA-prime/MVA-boost or Ad5-prime/MVA-boost protocols was highly immunogenic resulting in significant IFN-γ ELISPOT responses by splenocytes against recombinant vaccine antigens and a range of HAV specific peptides. This included strong recognition of a T-cell epitope GFAEINPIA located near the C-terminus of the fusion protein. Antibody responses to recombinant vaccine antigens and HAV specific peptides but not GFAEINPIA, also occurred. No immune recognition of vaccine antigens occurred in any sham vaccinated *Mycobacterium avium* subspecies *paratuberculosis* infected mice. Vaccination using either protocol significantly attenuated pre-existing *Mycobacterium avium* subspecies *paratuberculosis* infection measured by qPCR in spleen and liver and the Ad5-prime/MVA-boost protocol also conferred some protection against subsequent challenge. No adverse effects of vaccination occurred in any of the mice**.**

**Conclusions/Significance:**

A range of modern veterinary and clinical vaccines for the treatment and prevention of disease caused by *Mycobacterium avium* subspecies *paratuberculosis* are needed. The present vaccine proved to be highly immunogenic without adverse effect in mice and both attenuated pre-existing *Mycobacterium avium* subspecies *paratuberculosis* infection and conferred protection against subsequent challenge. Further studies of the present vaccine in naturally infected animals and humans are indicated.

## Introduction

*Mycobacterium avium* subspecies *paratuberculosis* (MAP) is the causative organism of Johne's disease which can affect many animal species including primates [1]. Johne's disease is a systemic infection whose principal clinicopathological manifestation is chronic inflammation of the intestine [2], [3]. The disease ranges from pluribacillary to paucimicrobial with chronic granulomatous inflammation like Leprosy in humans [4]. Subclinical infection is widespread in farm animals especially dairy cattle [5] and there are wildlife reservoirs [6]–[8]. Infected animals secrete MAP in their milk [9] and excrete large numbers of these robust versatile pathogens onto pastures and the surrounding environment [10], [11] where they may survive for long periods [10], [12], [13] particularly within environmental protists [14].

Human populations are exposed to these multi-host pathogens in milk supplies [15] and are at risk from sources of environmental contamination and persistence [10], [11], [16]. In many regions Crohn's disease is increasing in incidence [17], [18] especially in children [19], [20]. MAP infection in humans is paucimicrobial with the organisms in a robust uniformly Ziehl Neelsen (ZN) negative phenotype and is difficult to detect [21]. However, when appropriate laboratory methods are used most people with chronic inflammation of the intestine of the Crohn's disease type are found to be infected with these proven chronic enteric pathogens [22]–[27]. MAP infections are difficult to eradicate [21].

Conventional veterinary vaccines against MAP have generally comprised killed organisms in oil injected subcutaneously in young animals [28]–[31]. Used in the field, these vaccines are effective in reducing the incidence of clinical disease [32]–[40] and attenuate pre-existing infection [41]. However, such whole killed vaccines do not eliminate subclinical MAP infection nor its persistence in its ecological niche in the gut. Furthermore, about half of the animals receiving whole killed MAP vaccines become false positive using the conventional tuberculin skin test diagnostic for bovine tuberculosis [42], [43]. Experimental MAP vaccines have comprised recombinant MAP antigens [44]–[50], expression library immunisation [51] and DNA vaccines expressing conserved mycobacterial proteins [52].

With the scale and complexity of the expanding problem of MAP infection in animals and the increasing evidence for the involvement of these multi- host pathogens in human disease [27], [53] there is a need for new candidate vaccines suitable for preventative and therapeutic use. Here we report the development of a vaccine against MAP comprising two secreted and two cell surface mycobacterial components incorporated in a fusion construct designated HAV and delivered in recombinant DNA, human Ad5 and MVA vectors. We describe the safety and immunogenicity of vaccination of C57BL/6 mice using DNA-prime/MVA-boost and Ad5- prime/MVA-boost and the efficacy of vaccination in therapeutic and prophylactic roles against experimental MAP infection.

## Results

### Full length expression of the predicted 95 kDa polypeptide HAV from recombinant vaccines in permissive cell lines

**Figure 1:**
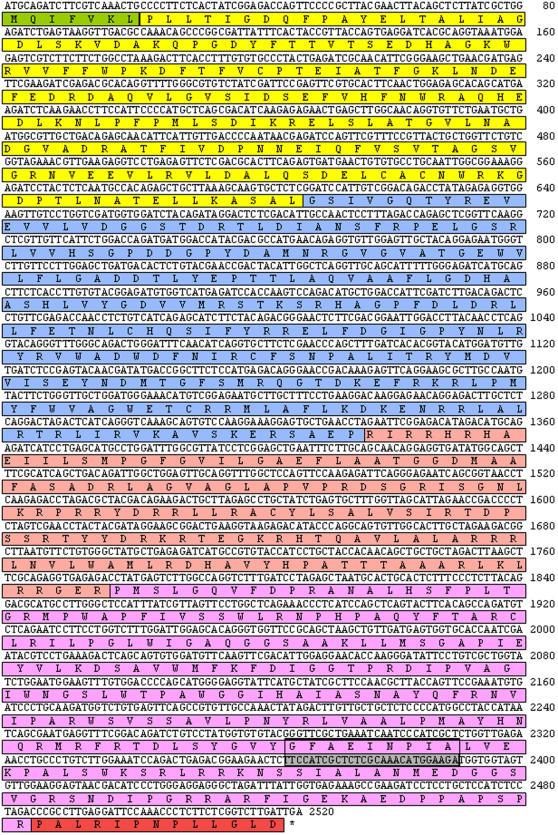
Structure and composition of the MAP vaccine fusion construct designated HAV. Coding and amino acid sequences of the four MAP antigen fusion protein HAV comprising AhpC MAP1589c (Yellow), Gsd MAP1234 (Blue), p12 MAP2444c (Pink), Mpa MAP1235 (Magenta), Ubiquitin leader (Green), pK C-terminal tag (Red). The strong murine T-cell epitope found in Mpa designated peptide F9.1 is boxed.

The assembly and insertion of the fusion construct HAV into DNA vaccine using the pSG2 vector and into the recombinant Ad5 and MVA viral vaccines is described in Materials in Methods. The HAV four antigen fusion protein (Figure 1) was seen to be expressed from pSG2.HAV as well as from MVA.HAV and Ad5.HAV when transfected or infected into the appropriate permissive cell lines. The antigen was visualized as the predicted 95 kDa polypeptide using western blots developed with anti-pK monoclonal antibody recognizing the C-terminal tag confirming full length expression (data not shown). Both MVA.HAV and Ad5.HAV recombinant clones were stable over 5 passages.

### Broad antigen specific T-cell responses without adverse effect in naïve C57BL/6 mice following DNA-prime/MVA-boost or Ad5-prime/MVA-boost vaccinations

**Figure 2:**
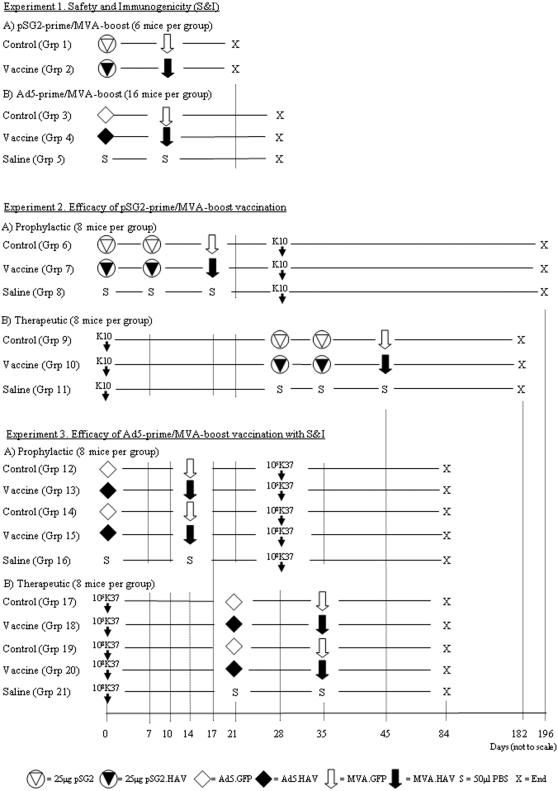
Protocols for vaccination studies. Viral doses were 10^8^ pfu per mouse. Routes of administration were i.m. for pSG2, i.v. for MVA boost in Experiments 1A and 2, and i.d.p. (intradermal into pinna) for Ad5 and MVA vaccination in Experiment 1B and Experiment 3.

**Figure 3:**
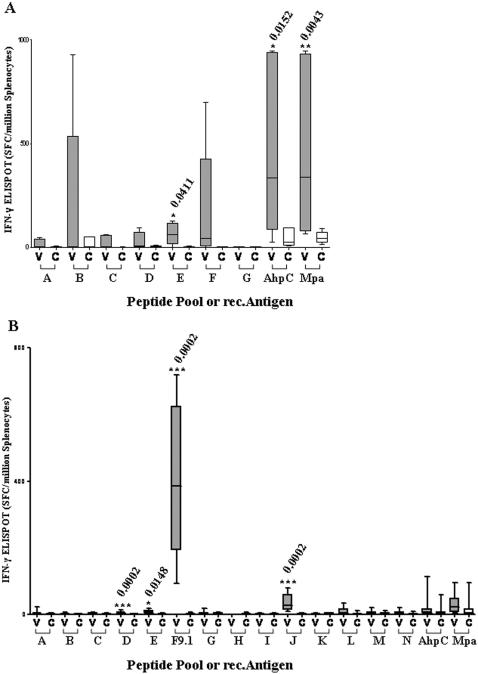
ELISPOT splenocyte responses. These include responses to peptide pools, selected peptide (F9.1), and recombinant vaccine antigens (AhpC, Mpa) in Control (Grp 1) mice (C) and Vaccine (Grp 2) mice (V), 4 weeks after immunisation A) using pSG2-prime/MVA-boost (Experiment 1A) or B) Ad5-prime/MVA-boost (Experiment 1B). Significant responses are indicated.

Vaccination protocols are summarised in Figure 2. Antigen-specific ELISPOT responses to recombinant proteins and peptides in vaccinated naïve mice compared with vector only control mice following immunization are shown in Figure 3. Three weeks after pSG2-prime/MVA-boost (Figure 2: Experiment 1A) there was significant recognition of rec.AhpC and rec.Mpa together with peptide pool E located in the amino-terminal portion of Mpa (Figure 3A). Increased recognition of peptide pool B and pool F was only seen in some mice and did not reach statistical significance. A different profile of antigen recognition was obtained using Ad5-prime/MVA-boost (Figure 2: Experiment 1B) in which there was no significant response to the recombinant antigens but highly significant recognition of peptide pool D (AhpC), pool E (Mpa), pool F (Mpa) and pool J (Gsd/p12) (Figure 3 B). The reactivity of pool F was found to be located in the single peptide designated F9.1 having the sequence GFAEINPIA. No adverse effects were observed in any of the 22 vaccinated naïve mice.

### DNA-prime/MVA-boost vaccination provides limited protection against subsequent MAP infection but significantly attenuates pre-existing infection without adverse effect

**Figure 4:**
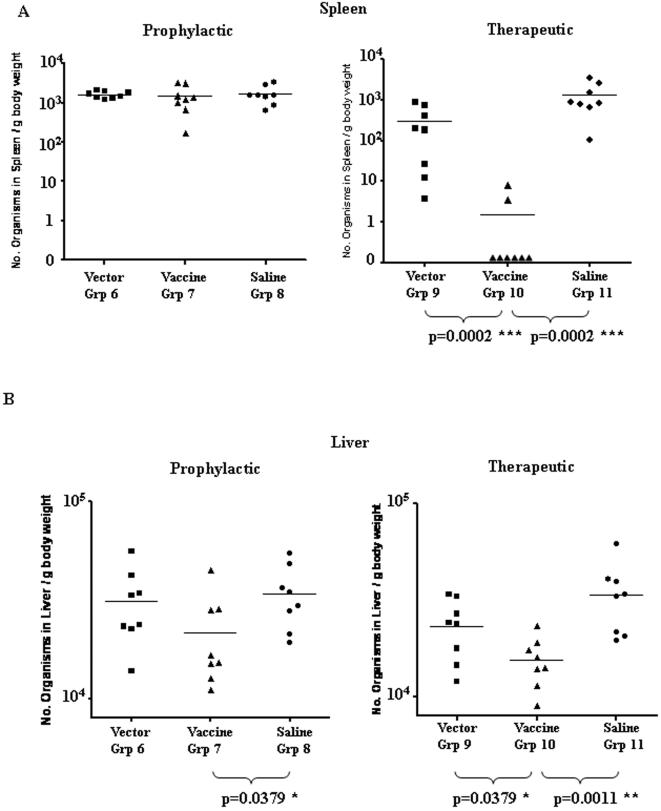
Quantitation of the tissue load of MAP. This was performed by qPCR in A) spleen and B) liver 28 weeks after prophylactic or therapeutic pSG2-prime/MVA-boost vaccination protocols in mice challenged i.p. with 10^7^ MAP K10(m). Significant responses are indicated.

The effect of prophylactic and therapeutic vaccination (Figure 2: Experiment 2) using pSG2-prime/MVA-boost protocols (Groups 6–11) on the tissue loads of MAP K10 (m) in spleen and liver are shown in Figure 4. The prophylactic vaccination protocol had no significant effect on the number of organisms in the spleens of the mice. There was a small but significant reduction of MAP load in the livers of vaccinated mice compared with the saline control group. After therapeutic vaccination however, MAP could not be detected in the spleens of 6 of the 8 mice in Group 10. In the remaining two mice the number of organisms was at the lower limit of detection by qPCR. The overall therapeutic effect was highly significant compared with vector only and saline control groups. Therapeutic vaccination also resulted in a small but significant reduction in the number of organisms in the livers of vaccinated animals compared with control groups. No adverse effects of vaccination were seen in any of the 16 vaccinated infected mice including those in the therapeutic protocol with an active MAP infection at the time of vaccination.

### Reproducible broad antigen specific T-cell responses to Ad5-prime/MVA-boost vaccination accompanied by significant prophylactic and therapeutic efficacy against a low and high level of MAP infection without adverse effect

**Figure 5:**
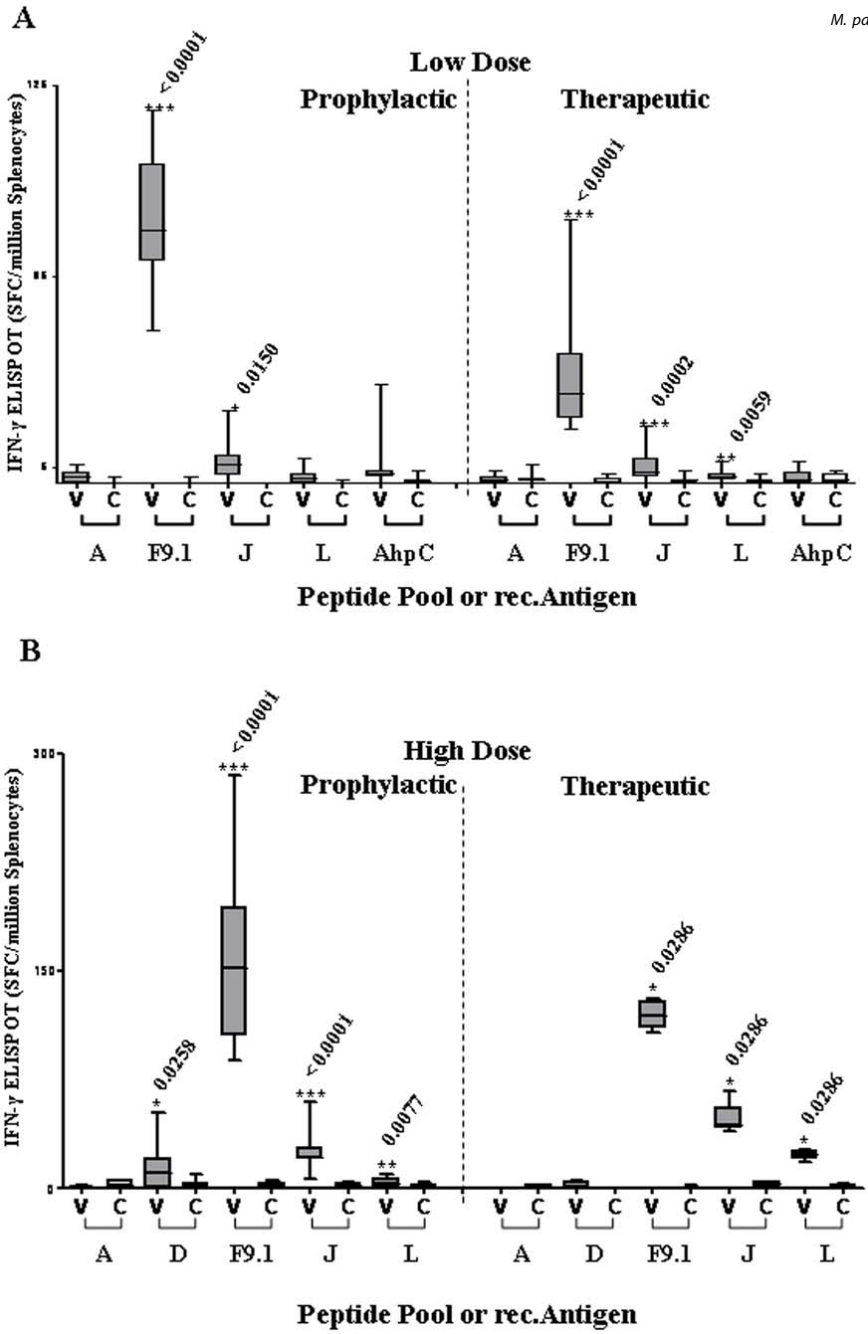
ELISPOT splenocyte responses. These include responses to peptide pools (A, J and L), the peptide epitope F9.1 (sequence GFAEINPIA) and rec.AhpC following prophylactic or therapeutic vaccination in A) low dose (10^5^) and B) high dose (10^8^) MAP K37 infection. C = Vector only control, V = Vaccine. Low dose prophylactic results correspond to Control (Grp12) and Vaccine (Grp13); Low dose therapeutic results correspond to Control (Grp17) and Vaccine (Grp18); High dose prophylactic results correspond to Control (Grp14) and Vaccine (Grp15); High dose therapeutic results correspond to Control (Grp19) and Vaccine (Grp20) as shown in Fig. 2.

ELISPOT responses to selected synthetic peptide antigens in mice infected with low dose (10^5^) MAP K37 following prophylactic and therapeutic protocols using Ad5-prime/MVA-boost (Figure 2: Experiment 3) are shown in Figure 5A. Significant recognition was seen with peptide F9.1 and pool J in both protocols with additional recognition of pool L (Gsd) in the therapeutic protocol. At high dose (10^8^) challenge there was also significant recognition of peptide F9.1, pool J and pool L in both prophylactic and therapeutic protocols. Pool D was also significantly recognized in the prophylactic experiment (Figure 5B). In addition, none of the recombinant HAV antigens nor any of the synthetic peptides were recognized in ELISPOT assays carried out on spleen cells from saline sham vaccinated MAP infected mice.

**Figure 6:**
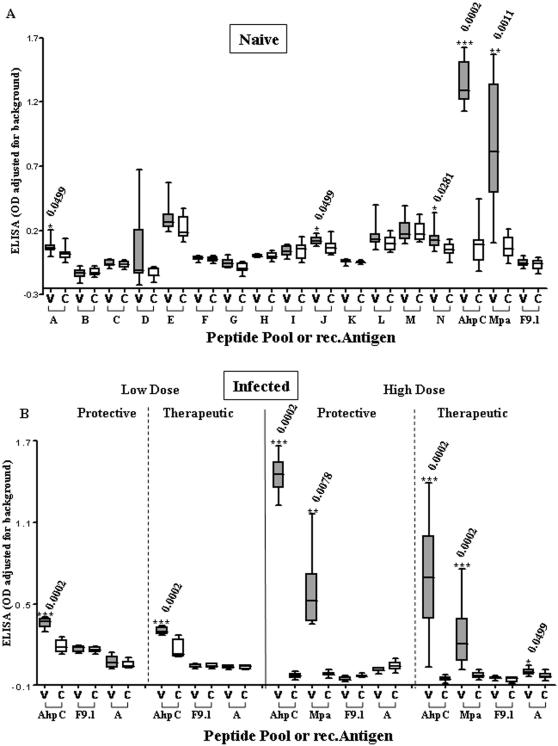
Antibody responses to vaccination. A) ELISA IgG_total_ responses in naïve uninfected mice, to peptide pools (A–N), the peptide epitope F9.1 (sequence GFAEINPIA) and recombinant vaccine antigens (AhpC, Mpa) 4 weeks after Ad5.HAV-prime/MVA.HAV-boost vaccination. C = Control (Grp3), V = Vaccine (Grp4) from Experiment 1B. B) ELISA IgG_total_ responses to peptide pool A, the peptide epitope F9.1 (sequence GFAEINPIA) and rec.AhpC following prophylactic or therapeutic vaccination in low dose (10^5^) and high dose (10^8^) MAP K37 infection. C = Vector only control, V = Vaccine. Low dose prophylactic results correspond to Control (Grp12) and Vaccine (Grp13); Low dose therapeutic results correspond to Control (Grp17) and Vaccine (Grp18); High dose prophylactic results correspond to Control (Grp14) and Vaccine (Grp15); High dose therapeutic results correspond to Control (Grp19) and Vaccine (Grp20) as shown in Fig. 2.

Specific IgG_total_ antibody responses to rec.Ahpc and rec.Mpa together with responses to all peptide pools and peptide F9.1, four weeks after vaccination of naïve mice using Ad5-prime/MVA-boost are shown in Figure 6A. Highly significant antibody responses to vaccination compared with vector only controls were seen with both recombinant HAV antigens. Significant antibody responses to vaccination were also observed with peptide pool A (AhpC), pool J (p12/Gsd) and pool N (Gsd). There was no antibody recognition of peptide F9.1.

The specific IgG_total_ antibody responses in low and high dose MAP infected Ad5-prime/MVA-boost vaccinated mice are shown in Figure 6B. In these studies incorporating prophylactic and therapeutic protocols, the antibody responses in Ad5-prime/MVA-boost vaccinated mice compared with vector only controls are shown for recombinant HAV antigens and selected peptides. There were highly significant specific antibody responses to recombinant HAV antigens in all studies. No antibody recognition of peptide F9.1 was observed in any of the mice. At the higher infective dose there was a weak response to peptide pool A (AhpC).

**Figure 7:**
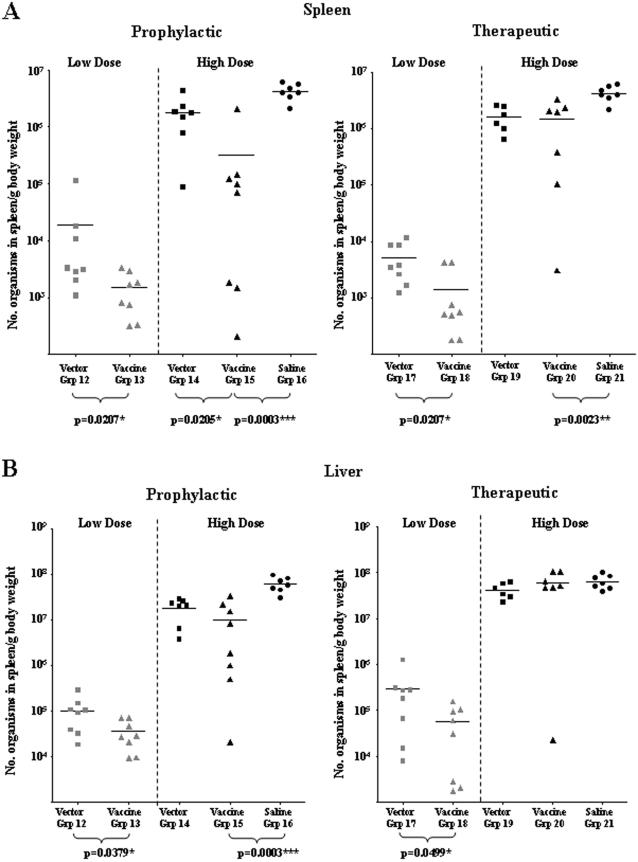
Quantitation of the tissue load of MAP. qPCR in A) spleen and B) liver 12 weeks after prophylactic or therapeutic Ad5-prime/MVA-boost vaccination protocols in mice challenged with either low dose (10^5^) or high dose (10^8^) MAP K37 (Experiment 3). Significant responses to vaccination are indicated.

The effect of prophylactic and therapeutic vaccination using Ad5-prime/MVA-boost protocols (Groups 12–21 Figure 2: Experiment 3) on the tissue loads of MAP K37 in spleen and liver are shown in Figure 7. Therapeutic vaccination after low dose MAP challenge resulted in a significant decrease in MAP load in the spleens (Figure 7A) and livers (Figure 7B). Therapeutic vaccination after high dose MAP challenge resulted in a significant decrease in MAP load in the spleens of vaccinated mice when compared with the saline only control Group 21 (Figure 7A). In one vaccinated animal in Group 20 there was a three log reduction in the MAP load in the liver which was not seen in the other animals. Prophylactic vaccination prior to low dose MAP challenge resulted in a significant decrease in MAP load in the spleens (Figure 7A) and livers (Figure 7B). The same result in spleen and liver occurred at high dose challenge and was statistically highly significant.

There were no significant differences in spleen weights or total body weights between vaccinated and control groups at low dose (10^5^) challenge with MAP K37 using the DNA-prime/MVA-boost immunization protocol. However the spleen weights, but not total body weights, of animals vaccinated with Ad5-prime/MVA-boost prior to high dose infection (Figure 2: Experiment 3A) were significantly lower than those in either vector only (p = 0.0003) or saline (p = 0.0205) sham vaccinated animals. No adverse effects due to therapeutic or prophylactic vaccination were seen in any naïve or MAP-infected animal.

## Discussion

In the present study we demonstrate that immunization of C57BL/6 mice using a fusion construct of four MAP genes delivered by differing routes in combinations of plasmid and viral vectors can achieve significant attenuation of pre-existing experimental MAP infection as well as some prophylaxis against subsequent challenge (Table 1).

**Table 1:** Summary of mean fold reductions in MAP loads in spleen and liver in response to vaccination

DNA-MVA	Therapeutic		Prophylaxic	
	Low	High	Low	High
Spleen	nd	894.0 (p = 0.0002)	nd	NS
Liver	nd	1.5 (p = 0.0379)	nd	2.2 (p = 0.0011)
Ad5-MVA				
Spleen	3.7 (p = 0.0207) *	2.9 (p = 0.0023)	12.8 (p = 0.0207) *	13.0 (p = 0.0003)
Liver	5.1 (p = 0.0499) *	NS	2.9 (p = 0.0379) *	5.9 (p = 0.0003)

nd = not done ; NS = not significant

* Comparison of vaccine and vector only

The 4 MAP genes within the 95 kDa polypeptide fusion construct were selected on the basis of known constitutive secreted expression or predicted presence at the mycobacterial cell surface. AhpC is a secreted virulence factor constitutively expressed in MAP and is shared by other pathogenic mycobacteria [54]. AhpC is up-regulated during transition of mycobacteria to a state of chronic persistence [55] and is involved in oxidative stress defence within host macrophages. Gsd is a predicted cell surface fucosyl transferase [56], [57] involved in peripheral oligosaccharide biosynthesis of serovar-2-specific glycopeptidolipid (GPL) in *M. avium* subsp. *avium*. However, the genes for the biosynthesis of the intermediate portion of *M. avium* subsp. *avium* GPLs between the lipopeptide core and peripheral oligosaccharides are not present in MAP and GPLs are reported to be absent from these chronic enteric pathogens [58]. The structure and functions of the acceptor substrates for peripheral fucosylation by Gsd in MAP have yet to be elucidated. Preliminary work in our lab using microarray analysis has suggested that *gsd* is up-regulated when MAP enters the intracellular environment.

p12 is the carboxyterminal 17 kDa fragment of p43 encoded by the MAP specific insertion sequence IS*900* [59]. This fragment is released into media from MAP cultures as well as from rec.*E.coli* expressing IS*900* [60] probably by proteolytic cleavage of membrane bound p43. Mpa is a putative acetyltransferase present together with Gsd in the pathogenicity associated GS element. The 10 predicted transmembrane regions within the structure of Mpa also suggest a pore function [61]. An intact Mpa gene is unique to MAP. It has homologues in *Shigella flexneri* and *Salmonella typhimurium* that are cell wall associated and determine serotype specificity and virulence [61]. Only the extracellular and intracellular loop regions of Mpa were included in the fusion construct to maximise the solubility of the fusion polypeptide removing hydrophobic transmembrane sequences. Stable full length expression of the 95 kDa fusion polypeptide HAV was demonstrated from all three vectors. Full length expression in vivo was evidenced by consistent ELISPOT recognition of the strong murine T cell epitope GFAEINPIA located near the C-terminal end of HAV.

The profiles of T-cell recognition of vaccine-specific recombinant antigen and synthetic peptides in ELISPOT assays were different between the DNA-prime/MVA-boost and Ad5-prime/MVA-boost vaccinated mice, probably reflecting differences in CD4+ and CD8+ responses to different vaccination protocols [62]. With plasmid priming in naïve mice (Figure 3A) there was T-cell recognition of both rec.AhpC and rec.Mpa with limited recognition of synthetic peptides. With Ad5.HAV-prime/MVA.HAV-boost in naïve mice (Figure 3B), no T-cell response to either recombinant antigen was seen but there was substantial recognition of synthetic peptides representing several domains throughout the fusion protein. This T-cell response to synthetic peptides was also seen in vaccination of MAP infected mice (Figure 5). Of particular note was the consistent identification of the strong murine T-cell epitope GFAEINPIA (peptide F9.1) sited near the carboxy-terminal end of the HAV polyprotein representing the fifth and smallest predicted extracellular peptide loop of Mpa [56]. It is interesting to note that T-cell reactivity to GFAEINPIA was a specific consequence of vaccination and did not occur in any sham vaccinated MAP infected mice.

By contrast, the Ad5.HAV-prime/MVA.HAV-boost protocol in naïve mice (Figure 6A) resulted in substantial IgG antibody recognition of rec.AhpC and rec.Mpa. This was also seen in response to vaccination in MAP infected animals (Figure 6B). Antibody responses to GFAEINPIA were consistently negative in all experiments. None of the recombinant proteins or synthetic peptides comprising the vaccine was recognised in any ELISPOT and ELISA tests on unvaccinated MAP infected animals.

MAP infection in unvaccinated mice caused substantial enlargement of the spleen. Prophylactic vaccination significantly reduced splenic enlargement following MAP challenge compared with vector only and saline control groups. However once splenic enlargement had been established by previous infection it was not reduced by subsequent vaccination within the 12 week time period of observation.

Natural MAP infection in animals can persist in a subclinical state for months or years. Despite the fact that many of the mice in the present study carried a huge infective load of these pathogens, none of the animals showed any visible deterioration in their clinical condition or significant diminution of their body weights even though the livers of some of them contained more than 10^8^ organisms. This illustrates the relatively benign host-pathogen relationships in experimental MAP infection in mice in sharp contrast to that seen with *M. tuberculosis*. It is consistent with the long recognised differences in pathogenicity and efficacy of anti-microbial drug treatments between MAP and *M. tuberculosis* infections in humans [63] the genetic basis for which is beginning to emerge [64].

There were substantial differences in the prophylactic and therapeutic effects of vaccination on the burden of MAP infection in spleen and liver between individual mice within groups. Despite this, both prophylactic and therapeutic vaccination protocols using the HAV insert delivered in both plasmid and viral vectors could achieve up to a 4 log reduction in the tissue abundance of these resilient and versatile intracellular pathogens. Experimental MAP infections in mice are not models of natural Johne's disease in ruminants or of MAP infection in Crohn's disease in humans. Although experimental infection in mice does involve the intestine and mesenteric lymph nodes the infection is general and disseminated [65]. Experimental infection using laboratory cultures of bovine strains of MAP in animals is uniformly pluribacillary with the organisms in their classical ZN-positive mycobacterial phenotype. In humans with Crohn's disease MAP infection is paucimicrobial with the organisms in a uniformly ZN-negative phenotype with accompanying florid granulomatous inflammatory disease similar to tuberculoid leprosy [66]. The ability of the fusion polypeptide HAV to confer some protection against challenge and attenuate pre-existing MAP infection when delivered in combinations of DNA or viral vectors suggests that further studies of the present vaccine in naturally infected animals and humans are indicated.

## Materials and Methods

### Vaccine construction

A 2526 bp fusion construct encoding a single polypeptide of 841 amino acids designated HAV (Figure 1) was assembled in pGEM®-T Easy (Promega, UK) from 40mer oligonucleotide precursors with 20mer overlaps as previously described [67]. The construct comprised four MAP genes fused in-frame, in the order MAP1589c (designated AhpC), MAP1234 (designated Gsd), MAP2444c (designated p12), and MAP1235 (designated Mpa). Oligos were designed with optimised mammalian codon usage. The 10 predicted hydrophobic transmembrane regions in MAP1235 and a lipid acylation motif in MAP1234 were excluded. The human ubiquitin leader sequence (MQIFVKL) was engineered in frame at the N-terminus of MAP1589c and a pK monoclonal antibody recognition sequence RIPNPLLGLD (Serotec, UK) at the C-terminus of MAP1235. Unique cloning sites were also included between each in-frame MAP ORF and at each end of the construct to facilitate cloning of each ORF into individual cloning vectors (pQE80 series, Qiagen, UK).

### Cloning of HAV into delivery vectors

**Figure 8:**
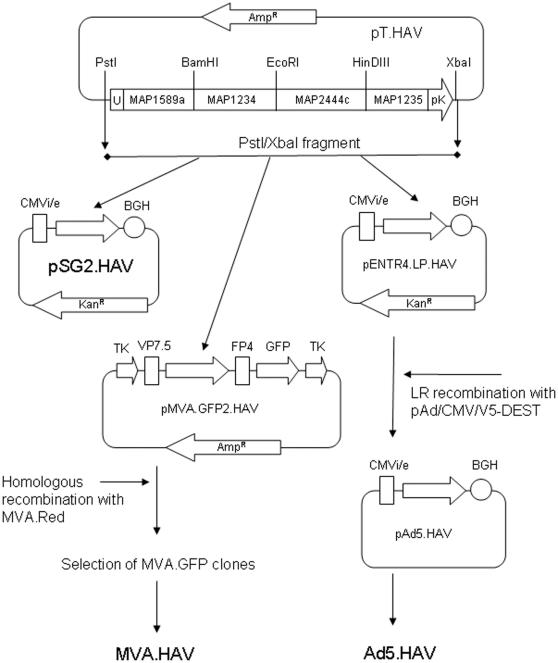
Strategy used for the insertion of HAV into pSG2 plasmid, Ad5 and MVA vectors.

The HAV construct was then introduced into delivery and shuttle vectors (summarised in Figure 8). Briefly, pSG2.HAV was constructed by blunt end cloning the *Pst*I/*Xba*I Havilah fragment of pT.HAV into the CMV intron A site of vector pSG2 [68] in which expression of HAV is driven by the upstream CMV intermediate/early promoter. Immediately downstream of the HAV insertion there was a BGH polyadenylation termination sequence (BGH). MVA.HAV was selected from GFP forming plaques visualized by fluorescence microscopy generated by recombination with MVA.Red infected into CEF cultures co-transformed with pMVA.GFP2.HAV constructed from the *Pst*I/*Xba*I Havilah fragment of pT.HAV inserted into the pMVA.GFP shuttle plasmid downstream of the Vaccinia virus P7.5 promoter (VP7.5) and upstream of green florescent protein (GPF) gene itself driven by the Fowlpox virus FP4b promoter (FP4), enclosed within an MVA thymidine kinase gene fragment (TK). Ad5.HAV was constructed by cloning *Pst*I/*Xba*I Havilah fragment of pT.HAV fragment from pT.HAV into the entry vector pENTR4.LS made by replacing the CMV promoter in Invitrogen pENTR4 with the CMV promoter and Intron A from pSG2. This resulted in a kanamycin resistant shuttle expression plasmid pENTR4.LP.HAV driven by CMVi/e. LR-recombination using the Virapower Adenovirus expression system kit (Invitrogen, UK) was then used to clone this expression cassette into the pAd/CMV/V5-DEST Gateway Vector to generate pAd5.HAV. 4 µg of *Pac*I digested pAd5.HAV was then transfected into HEK293A cells and plaques seen after 3 days were passaged twice, scraped into ice cold 10mM TrisHCl pH9.0 and stored as seed stocks at −80°C.

This resulted in a DNA vaccine (pSG2.HAV), a recombinant MVA vaccine (MVA.HAV) and a recombinant replication deficient human adenovirus type 5 vaccine (Ad5.HAV). Bulk preparations of pSG2.HAV transformed in *E.coli* JM109 (Promega, UK) were made using the Qiagen EndoFree Plasmid Mega Kit (Qiagen, UK). Bulk preparations of MVA.HAV were made from low passage MVA.HAV stock grown for 3 days in CEF cultures (SPF grade, IAH Compton, UK) as previously described [69]. Bulk preparations of Ad5.HAV were made from low passage Ad5.HAV seed stocks into large scale HEK293A cultures and purified using Adenopure Adenovirus Purification Kit (PureSyn Inc., USA). Recombinant viruses were titred to 10^10^ pfu/ml and stored at −80°C.

### Expression of the vaccine construct by plasmid and viral vectors

Plasmid DNA vaccine pSG2.HAV (2 µg) was transfected into confluent chicken fibroblasts (DF1, LGC Promochem, UK) using Lipofectin in OPTIMEM-I (Invitrogen) according to manufacturers protocols and incubated overnight. Stock MVA.HAV was infected into DF1 cells and incubated until visible plaques were observed by GFP fluorescent microscopy (3–4 days). Stock Ad5.HAV was infected into HEK293a cells and incubated until visible plaques were observed by light microscopy (7 days). In each case, cells were harvested, lysed in urea buffer (8 M Urea, 0.1 M NaH2PO4, 0.01 M TrisHCl, 0.2% 2-ME, 0.05% Tween20 : pH8.0), western blotted against monoclonal anti-pK tag antibody (Serotec) and visualized by anti-mouse HRP (Serotec) and DAB reagents (Perbio Science, UK) using standard protocols.

### Preparation and purification of recombinant antigens and synthetic peptides

Expression plasmids for AhpC and Mpa, each with a ubiquitin lead sequence and a C-terminal pK tag, were constructed by cloning fragments from pT.HAV, into the N-terminal His-Tag vector, pQE80L (Qiagen). This resulted in constructs pQE80.AhpC and pQE80.Mpa each fused with a 6xHis tag followed by the ubiquitin leader sequence at their N-termini and the pK–tag monoclonal epitope at their C-termini. Recombinant antigens were extracted using His-tagged selection from sonicated 30°C overnight culture lysates washed in 50 mM TrisHCl (pH 7.5) containing 1 mM PMSF onto Ni-Chelate agarose beads, eluted into PBS and stored at 4°C. Antigen concentrations were measured by absorbance at 280 nm and checked for purity and predicted size (rec.AhpC 26.8 kDa, rec.Mpa: 29.7 kDa) by PAGE and Western blotting using anti pK-tag monoclonal antibody (data not shown). A library of 142 overlapping mostly 15 residue synthetic peptides spanning the entire sequence of HAV was purchased from Alta Bioscience, UK. Peptides were combined in 14 pools A-N. Pool A-D corresponded to AhpC, pool E-G to Mpa, pool H-J to p12 and pool J-N to Gsd.

### IFN-γ ELISPOT assay and ELISA

For ELISPOT assay, the proportion of IFN-γ secreting splenocytes in 1×106 fresh spleen cell preparations after overnight stimulation with test peptides or recombinant *M. paratuberculosis* antigens (final concentration of 2.5 µg/ml) was determined as previously described (70). For ELISA, test peptides or recombinant MAP antigens were diluted in carbonate coating buffer (0.15 M Na2CO3, 0.35 M NaHCO3: pH 9.6) to a final concentration of 2.5 µg/ml and plates (Maxisorp, Nunc, Denmark) coated overnight at 4°C. Plates were then washed twice in PBST (0.02% Tween 20 in PBS) and then blocked (1% BSA in PBST) for 2 hrs at room temperature. After 4 further washes in PBST, 100 µl of mouse serum diluted 1∶200 in PBS were added and incubated for 1 hr at room temperature. Plates were again washed 4 times in PBST and then incubated for 1 hr with F(ab2′) rabbit anti-mouse-HRP conjugated IgGtotal (Serotec) diluted 1∶1000 in blocking buffer. After 4 PBST washes and one PBS wash, plates were developed with TMB (Perbio Science).

### Quantitative IS*900* PCR (qPCR)

Because of its extremely slow growth or lack of growth in culture and the impracticability of the reliable quantification of MAP in tissues by CFU the tissue load of these organisms in infected mice was measured by qPCR. Duplicate tissue samples (6–10 mg) from spleen and liver obtained at autopsy were washed in Red Cell Lysing Buffer then ground with a sterile plastic pestle (Techmate, UK). Samples were centrifuged at 12,000×g for 15 minutes at room temperature and then digested for 2 hrs at 37°C in 600 µl mycobacterial lysis buffer (MLB: 2 mM NaEDTA, 400 mM NaCl, 10 mM TrisHCl pH 8.0, 0.2 µm filtered 0.6% SDS, 33 µg/ml Proteinase K) then mechanically disrupted in the FastPrep Ribolyser (Qbiogene) at a setting of 6.5 ms-2 for 45 s then immediately chilled again on ice for 15 min. After phenol/chloroform extraction, the DNA was precipitated at −20°C overnight with ethanol, the resulting pellets were washed in 70% ethanol and dissolved in TEx1 at 4°C overnight.

Samples (5 µl) were tested in duplicate by qPCR in the following reaction mix: 25 µl 2× ABsolute™ qPCR dUTP reaction mix (ABgene, UK), 1 U Uracil-DNA glycosylase (Roche), 0.2 µl (final conc. 0.4 µM) forward primer TJ50 (CAGCGGCTGCTTTATATTCC), 0.2 µl (final conc. 0.4 µM) reverse primer TJ51 (GCAGAGGCTGCAAGTCGT) and 0.1 µl (final conc. 0.8 µM) 5′-Fam:3′-BHQ1 labelled internal probe TJ52P (CAAAGACGCTGCGATCATC) made to 50 µl final volume with dH2O. Reactions were pre-treated at room temperature for 10 minutes with uracil-DNA glycosylase, then amplified using 1 cycle [94°C: 15 mins], 40 cycles [94°C: 30 secs, 58°C: 30 secs, 60°C: 1 min] collecting at 60°C. Quantification of IS*900* copy number was calibrated using a 10 fold dilution curve of pIDL60 stock solutions. Calculations of organism concentration per sample were made assuming that all MAP strains tested contained 17 copies of IS*900*.

### *M. avium* subspecies *paratuberculosis* strains

DNA-prime/MVA-boost vaccination experiments used a laboratory maintained strain of bovine MAP K10 (m). For subsequent Ad5-prime/MVA-boost experiments we used a bovine strain MAP K37 recently isolated from a Johne's disease cow in South Wales, UK. To facilitate the emulsification of inocula, MAP cultures were grown on SIDI medium (4.7 g Middlebrooks 7H10, 1.25 g D/L Asparagine, 1.25 g Casein Digest, 2 ml Glycerol, 0.02% Malachite Green, 7.5 g Agar Noble, 3 organic egg yolks, 50 mls OADC (Sigma, UK), 50 mls FC(B)S (Gibco, UK), 1 mg Mycobactin J , 25 mg Amphotericin B, 25 mg Naladixic Acid, 25 mg Vancomycin ; per 400 mls dH_2_O (pH 5.9)) at 37°C. Inocula were prepared fresh by scraping 12 week cultures (maximum 3 subcultures) into 2 mls sterile PBS and washing twice in PBS with vigorous passage through a 20G needle. Bacterial concentrations were determined by IS*900* qPCR and diluted in endotoxin-free PBS to 5×10^9^ organisms/ml just prior to inoculation.

### Vaccination protocols

Three experiments including 21 protocols investigating safety and immunogenicity (S&I) (Experiment 1), prophylactic and therapeutic efficacy of pSG2-prime/MVA-boost against MAP K10 challenge (Experiment 2) and prophylactic and therapeutic efficacy of Ad5-prime/MVA-boost with S&I against K37 challenge (Experiment 3) were performed (Figure 2). All experiments used 4–6 week old female C57BL/6 mice which were isolator maintained under containment category 3 conditions. Their physical condition was monitored daily and body weights recorded every two weeks. Mice were settled in for at least 7 days prior to experimentation. Intramuscular (50 µl i.m.) and tail vein intravenous (100 µl i.v.) vaccinations did not require anaesthesia. Intradermal vaccinations into the pinna of the ear (50 µl i.d.p.) were administered under general anaesthesia (2 parts water, 1 part Hypnorn, 1 part Hypnovel (Roche)) at 0.1 ml/10 g body weight. MAP was given in 200 µl buffer intraperitoneally (i.p.). Studies were performed under Home Office License PPL 70/5378. At the end of each study mice were killed by a Home Office approved humane procedure. Outcome measures were the clinical condition and body weights of the mice together with autopsy appearances and spleen weights. Spleen cells and serum were taken for ELISPOT and ELISA testing. Efficacy of vaccination was assessed by measuring the abundance of MAP in spleen and liver tissue samples using qPCR as described. Five mice from unvaccinated control groups and one mouse from a vaccinated group were lost throughout the period of these studies from causes unrelated to vaccination.

### Statistics

The Mann-Whitney test was used throughout to test for significance in the ELISPOT and ELISA data and the tissue load of MAP between groups of individual mice.

## References

[1] Chacon O, Bermudez LE, Barletta RG (2004). Johne's disease, inflammatory bowel disease, and *Mycobacterium paratuberculosis*.. Ann Rev Microbiol.

[2] Buergelt CD, Hall C, McEntee K, Duncan JR (1978). Pathological evaluation of paratuberculosis in naturally infected cattle.. Vet Pathol..

[3] Clarke CJ, Little D (1996). The pathology of ovine paratuberculosis: gross and histological changes in the intestine and other tissues.. J Comp Pathol.

[4] Clarke CJ (1997). The pathology and pathogenesis of paratuberculosis in ruminants and other species.. J Comp Pathol.

[5] Manning EJ, Collins MT (2001). *Mycobacterium avium* subsp. *paratuberculosis*: pathogen, pathogenesis and diagnosis.. Rev Sci Tech.

[6] Greig A, Stevenson K, Henderson D, Perez V, Hughes V (1999). Epidemiological study of paratuberculosis in wild rabbits in Scotland.. J Clin Microbiol.

[7] Raizman EA, Wells SJ, Jordan PA, DelGiudice GD, Bey RR (2005). *Mycobacterium avium* subsp. *paratuberculosis* from free-ranging deer and rabbits surrounding Minnesota dairy herds.. Can J Vet Res.

[8] Corn JL, Manning EJ, Sreevatsan S, Fischer JR (2005). Isolation of *Mycobacterium avium* subsp. *paratuberculosis* from free-ranging birds and mammals on livestock premises.. Appl Environ Microbiol.

[9] Sweeney RW, Whitlock RH, Rosenberger AE (1992). *Mycobacterium paratuberculosis* cultured from milk and supra mammary lymph nodes of infected asymptomatic cows.. J Clin Microbiol.

[10] Pickup RW, Rhodes G, Arnott S, Sidi-Boumedine K, Bull TJ (2005). *Mycobacterium avium* subsp. *paratuberculosis* in the catchment area and water of the River Taff in South Wales, United Kingdom, and its potential relationship to clustering of Crohn's disease cases in the city of Cardiff.. Appl Environ Microbiol.

[11] Pickup RW, Rhodes G, Bull TJ, Arnott S, Sidi-Boumedine K (2006). *Mycobacterium avium* subsp. *paratuberculosis* in lake catchments, in river water abstracted for domestic use, and in effluent from domestic sewage treatment works: diverse opportunities for environmental cycling and human exposure.. Appl Environ Microbiol..

[12] Riemann HP, Abbas B (1983). Diagnosis and control of bovine paratuberculosis (Johne's disease).. Adv Vet Sci Comp Med.

[13] Whittington RJ, Marsh IB, Reddacliff LA (2005). Survival of *Mycobacterium avium* subsp. *paratuberculosis* in dam water and sediment.. Appl Environ Microbiol.

[14] Mura M, Bull TJ, Evans H, Sidi-Boumedine K, McMinn L (2006). Replication and Long-Term Persistence of Bovine and Human Strains of *Mycobacterium avium* subsp. *paratuberculosis* within *Acanthamoeba polyphaga*.. Appl Environ Microbiol.

[15] Ellingson JL, Anderson JL, Koziczkowski JJ, Radcliff RP, Sloan SJ (2005). Detection of viable *Mycobacterium avium* subsp. *paratuberculosis* in retail pasteurized whole milk by two culture methods and PCR.. J Food Prot.

[16] Whittington RJ, Marshall DJ, Nicholls PJ, Marsh IB, Reddacliff LA (2004). Survival and dormancy of *Mycobacterium avium* subsp. *paratuberculosis* in the environment.. Appl Environ Microbiol.

[17] Gearry RB, Richardson A, Frampton CM, Collett JA, Burt MJ (2006). High incidence of Crohn's disease in Canterbury, New Zealand: results of an epidemiologic study.. Inflamm Bowel Dis.

[18] Vind I, Riis L, Jess T, Knudsen E, Pedersen N (2006). Increasing incidences of inflammatory bowel disease and decreasing surgery rates in Copenhagen City and County, 2003-2005: a population-based study from the Danish Crohn colitis database.. Am J Gastroenterol.

[19] Hildebrand H, Finkel Y, Grahnquist L, Lindholm J, Ekbom A (2003). Changing pattern of paediatric inflammatory bowel disease in northern Stockholm 1990–2001.. Gut.

[20] Turunen P, Kolho KL, Auvinen A, Iltanen S, Huhtala H (2006). Incidence of inflammatory bowel disease in Finnish children, 1987–2003.. Inflamm Bowel Dis.

[21] Hermon-Taylor J, Bull TJ, Sheridan JM, Cheng J, Stellakis ML (2000). Causation of Crohn's disease by *Mycobacterium avium* subspecies *paratuberculosis*.. Can J Gastroenterol.

[22] Bull TJ, McMinn EJ, Sidi-Boumedine K, Skull A, Durkin D (2003). Detection and verification of *Mycobacterium avium* subsp. *paratuberculosis* in fresh ileocolonic mucosal biopsy specimens from individuals with and without Crohn's disease.. J Clin Microbiol.

[23] Naser SA, Ghobrial G, Romero C, Valentine JF (2004). Culture of *Mycobacterium avium* subspecies *paratuberculosis* from the blood of patients with Crohn's disease.. Lancet.

[24] Autschbach F, Eisold S, Hinz U, Zinser S, Linnebacher M (2005). High prevalence of *Mycobacterium avium* subspecies *paratuberculosis* IS*900* DNA in gut tissues from individuals with Crohn's disease.. Gut.

[25] Romero C, Hamdi A, Valentine JF, Naser SA (2005). Evaluation of surgical tissue from patients with Crohn's disease for the presence of *Mycobacterium avium* subspecies *paratuberculosis* DNA by in situ hybridization and nested polymerase chain reaction.. Inflamm Bowel Dis.

[26] Sechi LA, Scanu AM, Molicotti P, Cannas S, Mura M (2005). Detection and Isolation of *Mycobacterium avium* subspecies *paratuberculosis* from Intestinal Mucosal Biopsies of Patients with and without Crohn's Disease in Sardinia.. Am J Gastroenterol.

[27] Feller M, Huwiler K, Stephan R, Altpeter E, Shang A (2007). *Mycobacterium avium* subspecies *paratuberculosis*: a systemic review and meta-analysis.. Lancet Infect Dis.

[28] Stuart P (1965). Vaccination against Johne's disease in cattle exposed to experimental infection.. Br Vet J.

[29] Sigurdsson B (1960). A killed vaccine against paratuberculosis (Johne's disease) in sheep.. Am J Vet Res.

[30] Wilesmith JW (1982). Johne's disease: a retrospective study of vaccinated herds in Great Britain.. Br Vet J.

[31] Larsen AB, Moyle AI, Himes EM (1978). Experimental vaccination of cattle against paratuberculosis (Johne's disease) with killed bacterial vaccines: a controlled field study.. Am J Vet Res.

[32] Saxegaard F, Fodstad FH (1985). Control of paratuberculosis (Johne's disease) in goats by vaccination.. Vet Rec.

[33] Kormendy B (1994). The effect of vaccination on the prevalence of paratuberculosis in large dairy herds.. Vet Microbiol.

[34] Wentink GH, Bongers JH, Zeeuwen AA, Jaartsveld FH (1994). Incidence of paratuberculosis after vaccination against *M. paratuberculosis* in two infected dairy herds.. Zentralbl Veterinarmed B.

[35] van Schaik G, Kalis CH, Benedictus G, Dijkhuizen AA, Huirne RB (1996). Cost-benefit analysis of vaccination against paratuberculosis in dairy cattle.. Vet Rec.

[36] Corpa JM, Perez V, Sanchez MA, Marin JF (2000). Control of paratuberculosis (Johne's disease) in goats by vaccination of adult animals.. Vet Rec.

[37] Kalis CH, Hesselink JW, Barkema HW, Collins MT (2001). Use of long-term vaccination with a killed vaccine to prevent fecal shedding of *Mycobacterium avium* subsp *paratuberculosis* in dairy herds.. Am J Vet Res.

[38] Begg DJ Griffin JF (2005). Vaccination of sheep against *M. paratuberculosis*: immune parameters and protective efficacy.. Vaccine.

[39] Reddacliff L, Eppleston J, Windsor P, Whittington R, Jones S (2006). Efficacy of a killed vaccine for the control of paratuberculosis in Australian sheep flocks.. Vet Microbiol.

[40] Singh SV, Singh PK, Singh AV, Sohal JS, Gupta VK, Vihan VS (2007). Comparative efficacy of an indigenous 'inactivated vaccine' using highly pathogenic field strain of *Mycobacterium avium* subspecies *paratuberculosis* 'Bison type' with a commercial vaccine for the control of Capri-paratuberculosis in India.. Vaccine.

[41] Gwozdz JM, Thompson KG, Manktelow BW, Murray A, West DM (2000). Vaccination against paratuberculosis of lambs already infected experimentally with *Mycobacterium avium* subspecies *paratuberculosis*.. Aust Vet J.

[42] Mackintosh CG, Labes RE, Griffin JF (2005). The effect of Johne's vaccination on tuberculin testing in farmed red deer (Cervus elaphus).. N Z Vet J.

[43] Muskens J, van Zijderveld F, Eger A, Bakker D (2002). Evaluation of the long-term immune response in cattle after vaccination against paratuberculosis in two Dutch dairy herds.. Vet Microbiol.

[44] Mullerad J, Michal I, Fishman Y, Hovav AH, Barletta RG (2002). The immunogenicity of *Mycobacterium paratuberculosis* 85B antigen.. Med Microbiol Immunol (Berl).

[45] Mullerad J, Hovav AH, Nahary R, Fishman Y, Bercovier H (2003). Immunogenicity of a 16.7 kDa *Mycobacterium paratuberculosis* antigen. Microb Pathog..

[46] Shin SJ, Chang CF, Chang CD, McDonough SP, Thompson B (2005). In vitro cellular immune responses to recombinant antigens of *Mycobacterium avium* subsp. *paratuberculosis.*. Infect Immun.

[47] Dupont C, Thompson K, Heuer C, Gicquel B, Murray A (2005). Identification and characterization of an immunogenic 22 kDa exported protein of *Mycobacterium avium* subspecies *paratuberculosis.*. J Med Microbiol.

[48] Koets A, Hoek A, Langelaar M, Overdijk M, Santema W (2006). Mycobacterial 70 kD heat-shock protein is an effective subunit vaccine against bovine paratuberculosis.. Vaccine.

[49] Basagoudanava SH, Goswami PP, Tiwari V (2006). Cellular immune responses to 35 kDa recombinant antigen of *Mycobacterium avium paratuberculosis*.. Vet Res Commun.

[50] Rigden RC, Jandhyala DM, Dupont C, Crosbie-Caird D, Lopez-Villalobos N (2006). Humoral and cellular immune responses in sheep immunized with a 22 kilodalton exported protein of *Mycobacterium avium* subspecies *paratuberculosis*.. J Med Microbiol.

[51] Huntley JF, Stabel JR, Paustian ML, Reinhardt TA, Bannantine JP (2005). Expression library immunization confers protection against *Mycobacterium avium* subsp. *paratuberculosis* infection.. Infect Immun.

[52] Sechi LA, Mara L, Cappai P, Frothingam R, Ortu S (2006). Immunization with DNA vaccines encoding different mycobacterial antigens elicits a Th1 type immune response in lambs and protects against *Mycobacterium avium* subspecies *paratuberculosis* infection.. Vaccine.

[53] Scanu AM, Bull TJ, Cannas S, Sanderson JD, Sechi L (2007). *Mycobacterium avium* subspecies *paratuberculosis* infection in Irritable Bowel Syndrome and comparison with Crohn's and Johne's diseases: common neural and immune pathogenicity.. J Clin Microbiol.

[54] Olsen I, Reitan LJ, Holstad G, Wiker HG (2000). Alkyl hydroperoxide reductases C and D are major antigens constitutively expressed by *Mycobacterium avium* subsp. *paratuberculosis*.. Infect Immun.

[55] Voskuil MI, Visconti KC, Schoolnik GK (2004). *Mycobacterium tuberculosis* gene expression during adaptation to stationary phase and low-oxygen dormancy.. Tuberculosis (Edinb).

[56] Sheridan JM, Bull TJ, Hermon-Taylor J (2003). Use of bioinformatics to predict a function for the GS element in *Mycobacterium avium* subspecies *paratuberculosis*.. J Mol Microbiol Biotechnol..

[57] Tizard M, Bull T, Millar D, Doran T, Martin H (1998). A low G+C content genetic island in *Mycobacterium avium* subsp. *paratuberculosis* and *M. avium* subsp. *silvaticum* with homologous genes in *Mycobacterium tuberculosis*.. Microbiology.

[58] Eckstein TM, Belisle JT, Inamine JM (2003). Proposed pathway for the biosynthesis of serovar-specific glycopeptidolipids in *Mycobacterium avium* serovar 2.. Microbiology.

[59] Tizard ML, Moss MT, Sanderson JD, Austen BM, Hermon-Taylor J (1992). p43, the protein product of the atypical insertion sequence IS*900*, is expressed in *Mycobacterium paratuberculosis*.. J Gen Microbiol.

[60] Naser SA, Gillespie RF, Naser NA, El-Zaatari FA (1998). Effect of IS*900* gene of *Mycobacterium paratuberculosis* on *Mycobacterium smegmatis*.. Curr Microbiol.

[61] Bull TJ, Sheridan JM, Martin H, Sumar N, Tizard M (2000). Further studies on the GS element. A novel mycobacterial insertion sequence (IS1612), inserted into an acetylase gene (mpa) in *Mycobacterium avium* subsp. *silvaticum* but not in *Mycobacterium avium* subsp. *paratuberculosis*.. Vet Microbiol..

[62] Estcourt MJ, Letourneau S, McMichael AJ, Hanke T (2005). Vaccine route, dose and type of delivery vector determine patterns of primary CD8+ T cell responses.. Eur J Immunol.

[63] Sanderson JD, Hermon-Taylor J (1992). Mycobacterial diseases of the gut: some impact from molecular biology.. Gut.

[64] Marri PR, Bannantine JP, Golding GB (2006). Comparative genomics of metabolic pathways in *Mycobacterium* species: gene duplication, gene decay and lateral gene transfer.. FEMS Microbiol Rev.

[65] Chiodini RJ, Buergelt CD (1993). Susceptibility of Balb/c, C57/B6 and C57/B10 mice to infection with *Mycobacterium paratuberculosis*.. J Comp Pathol..

[66] Hermon-Taylor J, El-Zaatari F, Pedley S, Bartram J, Rees G, Dufour A, Cotruv J (2004). The *Mycobacterium avium* subspecies *paratuberculosis* problem and its relation to the causation of Crohn's disease..

[67] Withers-Martinez C, Carpenter EP, Hackett F, Ely B, Sajid M (1999). PCR-based gene synthesis as an efficient approach for expression of the A+T-rich malaria genome.. Protein Eng.

[68] McShane H, Brookes R, Gilbert SC, Hill AV (2001). Enhanced immunogenicity of CD4(+) T-cell responses and protective efficacy of a DNA-modified vaccinia virus Ankara prime-boost vaccination regimen for murine tuberculosis.. Infect Immun.

[69] Hanke T, Blanchard TJ, Schneider J, Hannan CM, Becker M (1998). Enhancement of MHC class I-restricted peptide-specific T cell induction by a DNA prime/MVA boost vaccination regime.. Vaccine.

[70] Hanke T, Schneider J, Gilbert SC, Hill AV, McMichael A (1998). DNA multi-CTL epitope vaccines for HIV and *Plasmodium falciparum*: immunogenicity in mice.. Vaccine.

